# Twelfth Annual Report of the Trustees of the State Lunatic Hospital at Worcester

**Published:** 1845-07

**Authors:** 


### REVIEWS.

---

Art. VI.—*Twelfth Annual Report of the Trustees of the State Lunatic Hospital at Worcester.* Boston: Dutton & Wentworth. 1844. pp. 112. 8vo.

The gratifying statement with which this Report opens is, that while the Hospital, during the past year, has been more crowded with patients than ever before, its operations generally have been more successful and beneficial, than in any former year. We much question if the history of any similar institution furnishes more to gladden the heart than the Lunatic Hospital of Massachusetts. Its whole administration from the beginning has been characterized by a spirit of such active, enlightened benevolence; its officers have been so prompt to apply all the improvements of the age, and so zealous in discovering yet better methods of treating the insane, that it has naturally become the admiration and the praise of all lands. This spirit of its officers has been nobly backed by the Legislature of the State, and since March 1843 the Hospital has been so enlarged as to accommodate one hundred and fifty more patients, and been supplied "with all the necessary furniture and other accommodations for the same."

"The whole number of patients that have been admitted into the Hospital from the beginning is 2013. The whole number that have been discharged, including those that have died, 1750. There remain now, at the end of the year, 263 patients. The number admitted the past year has been 236. The number discharged 228, of whom 124 have recovered and 15 have died. Leaving at the Hospital 8 more patients at the close of the year than at its commencement."

But, encouraging as is their report, great as have been the improvements made in this admirable charity, the Trustees conclude by saying that—

"Much yet remains to be done to carry out the designs of the benevolent founders of this institution. There is much room for the charitably disposed and affluent to do good. Many improvements remain yet to be made, and many distressed subjects to be relieved."

Our most fervent wish is, that the legislators of all the States could go through the Lunatic Hospital at Worcester, and then hear this language from these worthy and enlightened men. How forcibly would it fall upon their ears, as most of them remembered that the Commonwealths for which they made laws were without any retreat for the insane! If the officers of the Massachusetts Hospital find in that institution many deficiencies to be supplied, what is to be said of those States which have not yet taken the first steps to provide such an asylum? Our brethren ought to be up concerning this grave matter. They must open the eyes of Legislatures to the great sin of neglecting this duty. It is a shame, that any State in Christendom should be destitute of a house where the unhappy lunatic might be made secure and comfortable, and have all the chances of improved science for restoration to his reason. But we leave this subject for the interesting statistics of this Report. The following statement shows how necessary are the additions now being made to the buildings of the Hospital, and gives but too faithful a picture of what is the state of things all over our country:

"In twelve years nearly three hundred patients have been sent away from the Hospital, who ought to have been retained. A large proportion of these individuals are now living, some in jails, some in cages and dungeons, and many in poor houses, in a miserable conditiou, not so much from a disposition to neglect them as from the difficulty of taking care of them in places so illy provided with suitable means."

The subjoined table exhibits the forms in which insanity was exhibited in the various individuals, and the proportion of cases in the sexes:

CLASSIFICATION OF INSANITY.

| | Whole No. | No. of each Sex. | Curable. | Total Curable. |
|---|---|---|---|---|
| *Mania*, - - - - - | 999 | | | |
| Males, - - - - - | | 540 | 350 | |
| Females, - - - - | | 459 | 323 | 673 |
| *Melancholia*, - - - - | 702 | | | |
| Males, - - - - - | | 310 | 168 | |
| Females, - - - - | | 392 | 250 | 418 |
| *Dementia*, - - - - - | 225 | | | |
| Males, - - - - - | | 139 | 4 | |
| Females, - - - - | | 86 | 4 | 8 |
| *Idiots*, - - - - - - - | 12 | | | |
| Males, - - - - - | | 10 | | |
| Females, - - - - | | 2 | | |

In regard to the education of the idiotic, the following encouraging facts are given:

"In 1828, a school for the instruction of idiotic male patients was established in Paris in connection with the Bicetre, and the plan has been extended by M. Leuret and Voisir. A similar school was established at the Salpetriere for females, in 1831, by M. Falret. At the Lancaster Lunatic Asylum, and at the Hanwell Asylum in England, efforts are now making, on the same benevolent plan, to resuscitate minds impaired by disease, and to develop the mental powers of the congenital idiot to a considerable degree of usefulness. A similar effort has been made in a few of the institutions in this country, in all, it is said, with unexpected success. I have no doubt that such cases have too often been despaired of when patient continuance in effort might have been followed by favorable results. Such cases are not necessarily hopeless. The brain, after severe disease, may be left in an inactive state when no organic lesion has taken place to fatally impair its functions.

"Many cases of palsy are followed by the loss of memory of names of persons, things, and even language, which, though in old age, have been learned anew more or less perfectly, so at last, as to be quite useful again. Some individuals of this description have a sort of double consciousness, the condition of knowledge, alternating after successive attacks of disease. May there not be even greater probability of restoring an intellect prostrated by insanity in early life, where none of the faculties are wholly lost, though all are weakened and debilitated?"

We subjoin another table showing the causes of insanity, and circumstances connected with causes and predisposition to insanity.

| | | | |
|---|---|---|---|
| Intemperance, | 260 | Jealousy, | 6 |
| Ill health, | 318 | Fright, | 13 |
| Masturbation, | 139 | Palsy, | 16 |
| Domestic Afflictions, | 203 | Hereditary, or having insane ancestors or kindred, | 525 |
| Religious, | 173 | Periodical, | 381 |
| Property, | 107 | Homicidal, | 22 |
| Disappointed Affection, | 63 | Have cammitted Homicide, | 16 |
| Disappointed ambition, | 33 | Suicidal, | 213 |
| Epilepsy, | 49 | Have committed Suicide | 10 |
| Puerperal, | 56 | Arising from physical causes, | 807 |
| Wounds of the Head, | 23 | Arising from moral causes, | 604 |
| Abuse of Snuff and Tobacco, | 9 | | |
| Many not classed. | | | |

The causes as operating with different force on men in various occupations are shown in the following extract:

"Of the 160 farmers, 32½ per cent. became insane by intemperance, 14½ per cent. by the secret vice, 13¾ per cent. by religious influences, and 12½ per cent. by trouble respecting property.

"Of 91 laborers, 60 per cent. are from intemperance, 14 per cent. from the secret vice, 6½ per cent. from religious influences, and 13 per cent. from trouble about property.

"Of 46 seamen, 54 per cent. of the cases are from intemperance, 11 per cent. from the secret vice, 11 per cent. from religious influences, and 5½ per cent. from trouble about property.

"Of the 53 who pursue active mechanical trades, carpenters, blacksmiths, &c., 35 per cent. arise from intemperance,

13 from the secret vice, 11 from religious influences, and 13 from anxiety about property.

"Of the 155 who pursue light and sedentary employments, including merchants, printers, students, and shoemakers, 12 per cent. arise from intemperance, 50 per cent. from the secret vice, 9 from religious influences, and 11 from anxiety about property.

"Of 17 professional men who have been in the Hospital, 4 per cent. became insane by intemperance, 6 by the secret vice, 1 from religious influences, and 3 from anxiety about property."

*Per Cent of Recoveries compared with the Admitted.*

| | |
|---|---|
| "There have been in the Hospital 2013 patients, of whom 916 have recovered, which is - - - - - - - - - - - - - - - - - | 45½ per cent. |
| "There have been admitted to the Hospital the last year 236 patients, and there have recovered 124, which is - - - - - - - - | 52 per cent. |
| "There have been admitted 127 cases of duration less than one year, of these 93 have recovered, which is - - - - - - - - - - | 73 per cent. |

"The per cent. of recoveries of recent cases is very large in the American Institutions, which speak well for the correctness of the moral and medical management. The treatment of the insane in all the American Hospitals is on the same principles and the appliances very nearly alike in each. Some depend upon *medicine* more than others, but when it is prescribed, it is nearly the same in all."

Dr. Woodward, the untiring attendant, relates at considerable length the moral and medical treatment of the inmates of the institution. Although less professional than any other part of his interesting report, we give his account of the "*Religious Exercises*," and with it conclude this hasty notice, with an expression of our gratitude to this benefactor of his race.

"The religious services of the chapel have been conducted, the past year, by our Chaplain, the Rev. George Allen, with his usual fidelity and ability. He has not only officiated twice on each Sabbath, but has attended devotional services each evening in the Johonnot Hall, at which a large proportion of our whole family have usually been present. Reading a portion of Scripture, singing and prayer constitute this exercise.

"We feel that the experiment of religious services and instruction at this Hospital has been wholly favorable, and far more extensively useful than was at first anticipated. A larger class have been able to attend than was then expected, and the individuals whom we at first supposed might be excited and injured by these services, have been found to attend with entire tranquillity and composure.

"The influence of religious instruction at daily prayers, and weekly in the Chapel, has given our patients favorable impressions of the character and designs of the Hospital, and has increased their confidence in the good intentions of the officers.

"In many instances, religious instruction has left influences far above its moral effects on the management of the insane. It has made permanently good impressions upon the character of individuals, amending the heart, improving the life, awakening a sense of religious obligation, and transforming the habits from levity to sobriety, from dissoluteness to the proprieties of rational life.

"Religious instruction is here, as elsewhere, designed to strengthen and encourage us in the way of virtue, to regulate and guide the wayward, to awaken the hopes, and to afford comfort to the anxious and desponding, to calm and traquilize the agitated, to do us all present good and prepare us for an heavenly inheritance.

"A large portion of the insane are fully capable of appreciating these influences in all things but such as relate to their particular delusions. They know *right* from *wrong*, *good* from *evil*, and when hurried by passion or impulse into improprieties or mischief, as fully repent and regret the consequences of their errors as other individuals. If this be true, then surely they should come under all the influences that tend to disengage them from error, and guide them in the way of duty. With a motive strongly presented to them, they can control their feelings and govern their conduct. What can more effectually reach the main-spring of action in their minds than religious truth, presented in the right manner, and with the right spirit?"

